# Cryo-EM structure of trimeric *Mycobacterium smegmatis* succinate dehydrogenase with a membrane-anchor SdhF

**DOI:** 10.1038/s41467-020-18011-9

**Published:** 2020-08-25

**Authors:** Hongri Gong, Yan Gao, Xiaoting Zhou, Yu Xiao, Weiwei Wang, Yanting Tang, Shan Zhou, Yuying Zhang, Wenxin Ji, Lu Yu, Changlin Tian, Sin Man Lam, Guanghou Shui, Luke W. Guddat, Luet-Lok Wong, Quan Wang, Zihe Rao

**Affiliations:** 1 State Key Laboratory of Medicinal Chemical Biology, Frontiers Science Center for Cell Responses, College of Life Sciences, Nankai University, 300353 Tianjin, China; 2 Laboratory of Structural Biology, Tsinghua University, 100084 Beijing, China; 3 Shanghai Institute for Advanced Immunochemical Studies and School of Life Science and Technology, ShanghaiTech University, 201210 Shanghai, China; 4 National Laboratory of Biomacromolecules,omolecules, Institute of Biophysics, CAS, 100101 Beijing, China; 5 High Magnetic Field Laboratory, Chinese Academy of Sciences, 230031 Hefei, China; 6 Hefei National Laboratory of Physical Sciences at Microscale and School of Life Sciences, University of Science and Technology of China, 230027 Hefei, China; 7 State Key Laboratory of Molecular Developmental Biology, Institute of Genetics and Developmental Biology, CAS, 100101 Beijing, China; 8 School of Chemistry and Molecular Biosciences, The University of Queensland, Brisbane, 4072 QLD Australia; 9 Department of Chemistry, Inorganic Chemistry Laboratory, University of Oxford, South Parks Road, Oxford, OX1 3QR UK

**Keywords:** Cryoelectron microscopy, Biophysics, Drug discovery, Microbiology

## Abstract

Diheme-containing succinate:menaquinone oxidoreductases (Sdh) are widespread in Gram-positive bacteria but little is known about the catalytic mechanisms they employ for succinate oxidation by menaquinone. Here, we present the 2.8 Å cryo-electron microscopy structure of a *Mycobacterium smegmatis* Sdh, which forms a trimer. We identified the membrane-anchored SdhF as a subunit of the complex. The 3 kDa SdhF forms a single transmembrane helix and this helix plays a role in blocking the canonically proximal quinone-binding site. We also identified two distal quinone-binding sites with bound quinones. One distal binding site is formed by neighboring subunits of the complex. Our structure further reveals the electron/proton transfer pathway for succinate oxidation by menaquinone. Moreover, this study provides further structural insights into the physiological significance of a trimeric respiratory complex II. The structure of the menaquinone binding site could provide a framework for the development of Sdh-selective anti-mycobacterial drugs.

## Abstract (web summary)

Diheme-containing succinate:menaquinone oxidoreductases (Sdh) are members of the complex II superfamily. Here, the authors present the 2.8 Å cryo-EM structure of *Mycobacterium smegmatis* Sdh2, which reveals membrane-anchored SdhF as a component of the complex and they discuss the electron/proton transfer pathway in the Sdh2 trimer.

## Introduction

Cellular respiration is an essential feature in the metabolism of many living cells. During respiration, electrons from the compounds in food are transferred to terminal electron acceptors through the electron transport chain (ETC), which couples the translocation of protons across a membrane (either cellular or mitochondrial). This creates a transmembrane proton gradient for a variety of processes, such as the synthesis of ATP (adenosine triphosphate). Electrons enter the ETC through either respiratory complex I or complex II^1,2^.

Complex II is both a functional member of the oxidative phosphorylation pathway and the Krebs cycle, thus linking these two energy-harvesting processes. Specifically, its roles are to catalyze the reversible oxidoreduction of succinate and fumarate in a soluble domain, coupled to the reversible oxidoreduction of quinone and quinol in a membrane-spanning domain^3^. Members of the complex II superfamily are identified as succinate:quinone oxidoreductases (SQRs; or succinate dehydrogenases, SDHs) or quinol:fumarate oxidoreductases (QFRs; or fumarate reductases, FRDs) according to the preferred direction of reaction^3,4^. No matter the direction, SQRs and QFRs share a common architecture with a large soluble domain and a smaller integral-membrane domain. They are comprised of a soluble flavin-containing subunit, an iron-sulfur cluster-containing subunit, either one or two transmembrane subunits and either zero, one, or two *b*-type heme groups. As a result, the membrane-spanning region has been regarded as the distinct evolutionary origin. Thus, the complex II superfamily has been further divided into type A–F subfamilies according to the number of transmembrane polypeptides and *b*-type hemes within the membrane-spanning region^5^. Intriguingly, the X-ray crystal structures have demonstrated that the oligomerization state of the complex II protein differs both across the superfamily and within subfamilies with monomeric^6,7^, dimeric^8–10^, and trimeric^11^ states all observed.

The diheme-containing family of complex II, namely type A and B subfamilies, found in a wide range of bacteria, is of particular interest, because its members support transmembrane electron transfer^12^. This process may or may not be coupled to transmembrane proton transfer, depending on the species and the direction of the reaction catalyzed in vivo^13^. The proton-coupled electron transfer events of specific diheme QFRs have already been studied based on the X-ray crystal structures from *Wolinella succinogenes*^9,14,15^ and *Desulfovibrio gigas*^10^. In spite of major advances in understanding the electron/proton transfer in diheme-containing QFRs, little is known about the molecular mechanism in the diheme-containing SQRs. Diheme-containing SQRs generally perform menaquinone-linked succinate oxidation^12^, a thermodynamically unfavorable reaction^16^. It has been proposed that the endergonic reduction of menaquinone by succinate is driven by the electrochemical proton potential^17–19^. The diheme-containing SQRs, performing menaquinone‐dependent succinate oxidation, that have been isolated from *Thermus thermophiles*^20,21^, *Bacillus licheniformis*^18^, and *Corynebacterium glutamicum*^22^ exist in trimeric forms and exhibit positive cooperativity^20,21^. In addition, the diheme-containing SQR also plays pivotal cellular and energetic roles in mycobacteria, including *Mycobacterium smegmatis*^23^ and *Mycobacterium tuberculosis*^24,25^. Therefore, structural data for the diheme-containing SQRs can explain how electron–proton transfer is coupled in this thermodynamically unfavorable reaction. In the present study, we obtained a 2.8 Å cryo-electron microscopy (cryo-EM) structure of a trimeric form of an Sdh2 protein from *M. smegmatis*, which will help us to understand the mechanism of this catalytic process in the diheme-containing SQRs.

## Results and discussion

### Purification and characterization of *M. smegmatis* Sdh2

Most mycobacterial species including *M. smegmatis* harbor two putative genes encoding different SDHs designated Sdh1 and Sdh2^26^. Sdh2 has been reported to contain two *b*-type heme groups embedded in the membrane-bound domain^23^. To isolate the Sdh2 in a functional form, protein purification was performed according to the method described previously^27^. Nickel-nitrilotriacetic acid (Ni-NTA) affinity chromatography and gel filtration were used to obtain a purified supramolecular assembly (Supplementary Fig. 1a). All the expected components (subunits A, B, C, and D) of Sdh2 as well as an unknown component (subunit F, described below) were detected by sodium dodecyl sulfate-polyacrylamide gel electrophoresis (SDS-PAGE) (Supplementary Fig. 1b) and mass spectrometry (MS) (Supplementary Table 1). A single band on blue native PAGE (BN-PAGE) indicated that SDH is present (Supplementary Fig. 1c). Electronic absorption spectra and electron paramagnetic resonance (EPR) spectra showed signals that correspond to the prosthetic groups ([2Fe-2S], [4Fe-4S], [3Fe-4S], and two *b*-type heme groups), all required for electron transfer (Supplementary Fig. 1d, e). Sdh2 oxidized succinate with an apparent catalytic constant (*k*_cat_) of (3.16 ± 0.07) × 10^4^ s^−1^ and a Michaelis constant (*K*_m_) of 65.82 ± 6.46 μM (Supplementary Fig. 6). The data confirm that the purified Sdh2 is a functioning complex that couples succinate oxidation to menadione reduction.

### Structural overview

**Fig. 1:**
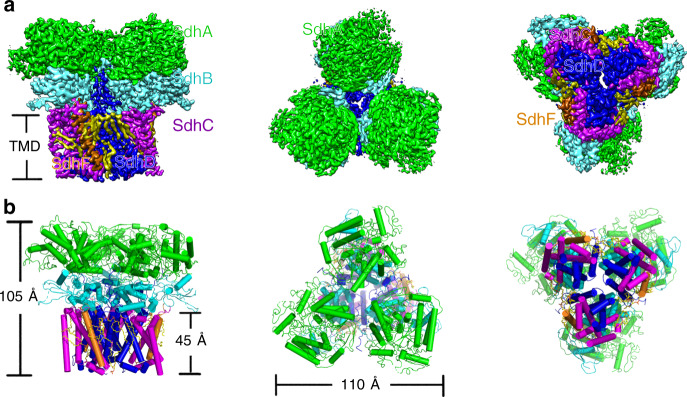
Overall architecture of the Sdh2 trimer from *M. smegmatis*. **a** Cryo-EM map of Sdh2 trimer at 2.8 Å resolution. Left: front view; middle: top view (from cytoplasm); right: bottom view (from periplasm). SdhA, SdhB, SdhC, SdhD, and SdhF subunits are colored in green, cyan, purple, blue, and orange individually. Lipids are colored in yellow. **b** Cartoon representation of Sdh2 trimer, using the same color scheme as above.

**Fig. 2:**
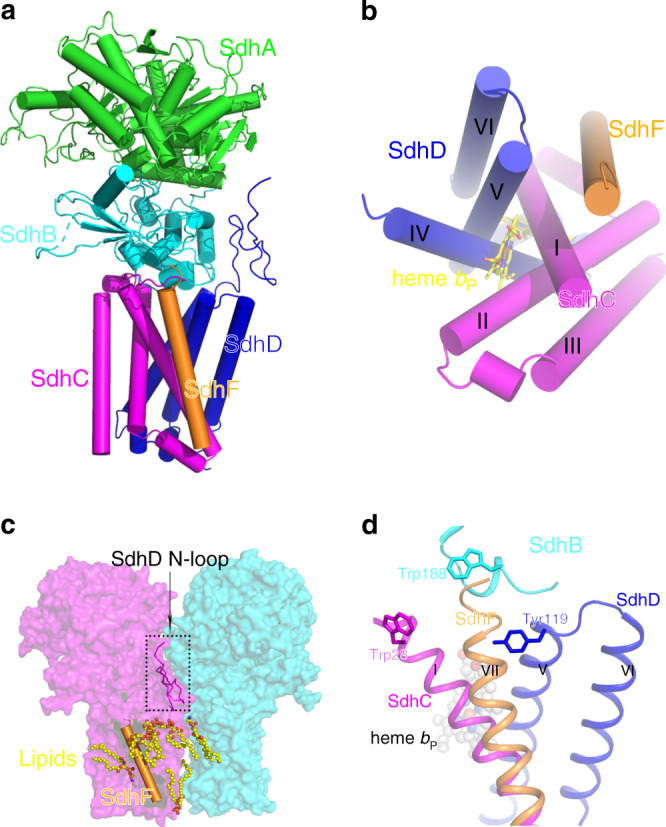
Location and interaction of SdhF subunit in Sdh2 protein. **a** One copy of the complex is composed of five subunits SdhA, SdhB, SdhC, SdhD, and SdhF, which are colored green, cyan, purple, blue, and orange, respectively. **b** Sequential display of transmembrane helices of the SdhC, SdhD, and SdhF subunits. **c** The distribution of phospholipids (shown in spheres) and SdhF subunit (shown in cylindric cartoon) and SdhD N-loop (shown in ribbons). **d** SdhF subunit is located in front of the conserved Q_P_-binding pocket.

The structure of Sdh2 was determined by cryo-EM to an overall resolution of 2.8 Å (Supplementary Figs. 2 and 3 and Supplementary Table 2). The dimensions of the Sdh2 trimer are 110 Å in width and 105 Å in height, adopting a mushroom-like shape (Fig. 1a). The form of trimeric assembly is similar to that observed in the *Escherichia coli* SQR^11^. In this structure, we observe extensive contacts between neighboring protomers of the trimer on both the cytoplasmic and membrane-spanning regions, suggesting that they are important for the assembly and stability of the trimer. Within the trimer, each of the three assemblies contains four canonical proteins: an FAD (flavin adenine dinucleotide)-binding protein (SdhA), an iron-sulfur protein (SdhB), and two membrane-anchored proteins (SdhC and SdhD), each with three transmembrane helices (Figs. 1b and 2a, b). Its subunit composition is similar to complex II from porcine and *E. coli* SQRs^6,11^. The SdhA subunit also can be divided into four domains^6,8^. Its capping domain is significantly disordered in the cryo-EM map. In other previously reported complexes, substrate^28^ and ligand^29^ binding has been shown to result in a change of orientation for this region. The SdhB subunit is shaped like a butterfly and organized into two domains. The SdhC and SdhD transmembrane helices exhibit a similar fold and are designated I to VI^8^ (Fig. 2b). The N terminus of SdhD subunit contains a long loop protruding into the cytoplasm (Fig. 2c), mediating the interactions between adjacent monomers in the assembly. In addition to the four known subunits of complex II, there is extra density corresponding to a transmembrane helix (Supplementary Fig. 3a), mainly located near the SdhC subunit (Fig. 2a, b). The density could be assigned to a previously undescribed subunit in the complex we identify as SdhF. Because of its much lower molecular weight, it was not detected on the SDS-PAGE. However, it could be detected by MS (Supplementary Table 1). We refer to it as SdhF because SdhE has already been used as an assembly factor of the SdhA subunit in SQR and QFR^30,31^. It is worth noting that the homologous sequences of SdhF subunit are mainly found in mycobacteria (Supplementary Fig. 5), which suggests that the analogs of Sdh2 complex might be widely distributed in mycobacteria.

All the prosthetic groups (FAD, [2Fe-2S], [4Fe-4S], [3Fe-4S], and two *b*-type heme groups) were unambiguously assigned into the cryo-EM map (Supplementary Fig. 3b). It has been previously shown that different residues in the FAD-binding site are responsible for either efficient SQR or QFR catalytic activity^32^. Analysis of the amino acids in *M. smegmatis* Sdh2 (e.g., key residues such as Gln-A48) shows it functions as an SDH^23^. The three different iron-sulfur clusters of SdhB subunit are coordinated by conserved cysteine residues observed previously in SQRs and QFRs^6,8,9,11^. Two *b*-type hemes, denoted as *b*_P_ heme and *b*_D_ heme based on their proximity to the hydrophilic subunits, are located in the membrane-spanning regions of the subunits SdhC and SdhD, and their axial ligands are His D107–His C87 and His D65–His C47, respectively. Indeed, the bis-histidine axial ligation is conserved throughout all complex IIs, regardless of the number of heme cofactors^6,8,9^.

Fifteen phospholipid molecules and six menaquinone/menaquinols are all clearly observed in the structure (Supplementary Figs. 3c and 4a), which is in agreement with MS (Supplementary Fig. 1f). However, no density for APM (acylated phosphatidylinositol mannoside) was observed, despite the fact that it was observed by MS. According to the distribution of lipids shown by MS, the proportion of APM was very low in comparison to other lipids in the sample. In the present structure, cardiolipin (CL), phosphatidylethanolamine (PE), phosphatidylinositol (PI), and phosphatidic acid (PA) molecules are mainly distributed in the cleft in the transmembrane space formed by neighboring subunits (Fig. 2c and Supplementary Fig. 3c), playing roles in mediating the interaction of subunits within protomers of the assembly and interactions between neighboring protomers of the assembly^27^.

### SdhF subunit blocks the canonical Q_P_-binding site

The subunit structure of complex II is highly conserved across bacteria, animals, and fungi, consisting of the four subunits SdhA, B, C, and D. However, other subunits have also been detected^30,33^, which play key roles in regulating the stability, activity, and incorporation of essential cofactors in many multi-subunit protein complexes in biogenesis^34^. Here, we have identified a 3 kDa subunit SdhF, which has only one transmembrane helix for its secondary structure, which is located near the transmembrane helix I of SdhC (Fig. 2a, b). SdhF subunit forms contacts with transmembrane helices I, V and SdhB subunit. There are also extensive interactions with molecules of PA, PE, PI, and CL (Fig. 2c and Supplementary Fig. 3c).

There are two quinone-binding sites observed in the structures of porcine SQR and *E. coli* QFR, which have been termed Q_P_ and Q_D_ sites^6,8^, located on opposite sides of the membrane-spanning region. In terms of the Q_P_ site, they are very strongly conserved between mitochondrial SQR and *E. coli* SQR^6,11^. This site is also well conserved in our structure (Supplementary Fig. 4b). However, the equivalent Q_P_-binding pocket in *M. smegmatis* Sdh2 structure is completely blocked by the SdhF subunit (Fig. 2d), making this site totally inaccessible from the outside. In addition, we could not find any other density that could be assigned as menaquinone in this binding pocket. Hence, we suggest that the Q_P_-binding site does not exist in the present structure, which is consistent with a previous study^35^.

### QD-binding site contributes to the structure-based drug discovery

**Fig. 3:**
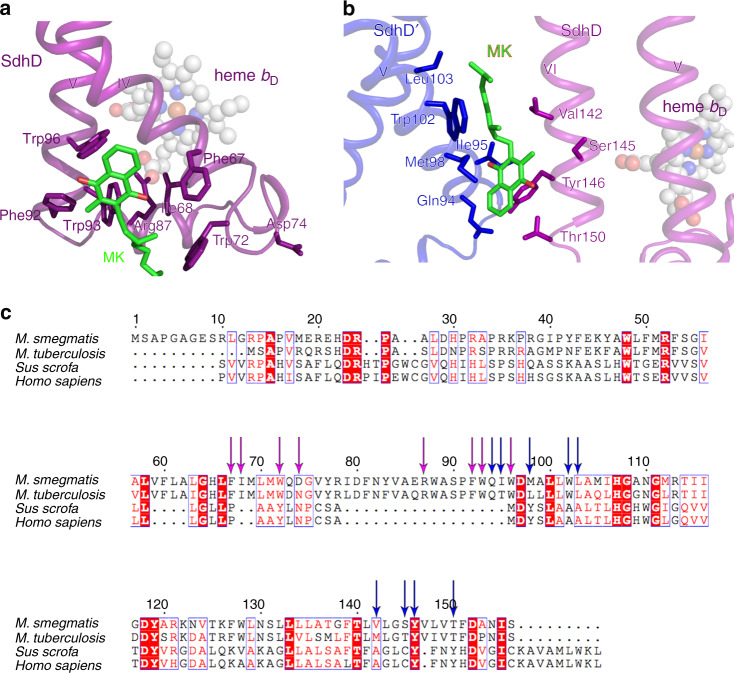
Quinone binding sites analysis in *M. smegmatis* Sdh2 trimer. **a** Close-up views of Q_D1_ binding site. The heme *b*_P_ nearby is shown in spheres. **b** Close-up views of Q_D2_ located between two neighboring SdhD subunits. **c** Sequence alignments of the SdhD subunits from *M. smegmatis*, *M. tuberculosis*, *Sus scrofa*, and *Homo sapiens*. Residues corresponding to Q_D1_ site and to Q_D2_ site are labeled with purple and blue arrows, respectively.

Quinone binding sites in respiratory complexes have varied sequences and specificities depending on the species of origin. They are of great interest, because they are often considered as clinical drug targets^27^. As mentioned above, there is no Qp binding site in *M. smegmatis* Sdh2. However, a Q_D_ binding site has been identified by means of bound inhibitor in porcine SQR^6^ (Supplementary Fig. 4c), which is termed the Q_D1_ binding site in our structure. In the porcine SQR, Trp-D134, Tyr-D61 (equivalent residues Trp-D88 and Tyr-D83 in our structure) and Lys-D135, Asp-D69 (equivalent residues Arg-D87 and Asp-D74 in present structure) in this pocket are considered to confer specificity for quinone binding and take the functions of quinone protonation, respectively. Coincidentally, we also identified a menaquinone close to the predicted Q_D_-binding pocket considered analogous to that in porcine SQR (Fig. 3a). It is surrounded by the side chains of residues Phe-D67, Ile-D68, Trp-D72, Phe-D92, Trp-D93, and Trp-D96. The edge-to-edge distance from MK to *b*_D_ heme is 15 Å, exceeding the 14 Å limit for physiological electron transfer^36^, and there are no observed hydrogen bonds to the carbonyl groups of MK, which are needed to couple proton abstraction and the accepting of electrons. Besides that, a salt bridge is observed between Arg-D87 and the ring C-propionate of *b*_D_ heme, indicating that the *b*_D_ heme is in oxidative status in the present structure^37^. Thus, we speculate that the endogenous menaquinone, close to Q_D1_-binding pocket, might be a representation of the reduced product MKH_2_, which is leaving the Q_D1_ site.

We also assigned a second bound MK/MKH_2_ to the cryo-EM density map adjacent to the *b*_D_ heme. Henceforth, we refer to this as the Q_D2_ site (Supplementary Fig. 4a). This binding site is located within the interface formed by membrane-spanning region of adjacent protomers in the assembly. This is also suggested in other studies, which showed that there is a quinone-binding site located between adjacent monomers in the trimeric Sdh complex from *T. thermophiles*^20,21^. The bound MK/MKH_2_ interacts with the side chains of Gln-D94, Ile-D95, Met-D98, Trp-D102, and Leu-D103 of transmembrane helix V in one protomer, and the Val-D142, Ser-D145, Tyr-D146, Val-D149, and Thr-D150 of transmembrane helix VI in an adjacent protomer of the assembly (Fig. 3b). As mentioned above, based on the relationship of proton abstraction and accepting electrons, MK should bind deeper close to polar residues, such as Gln-D94, Tyr-D146, and Thr-D150. Interestingly, the edge-to-edge distances between MK/MKH_2_ and the neighboring *b*_D_ hemes are 9 and 11 Å, respectively. These findings suggest that the Q_D2_ site can receive the electrons from one or two of the *b*_D_ hemes in adjacent protomers, and the bound molecule in the present structure is a representation of fully reduced MK. According to the kinetic characterization of the Q_D1/2_ mutants of Sdh2 using menadione/DCIP (succinate-2,6-dichlorophenolindophenol) assay (Supplementary Fig. 6), the *k*_cat_ values of the Q_D1_ and Q_D2_ sites are (2.16 ± 0.04) × 10^4^ and (1.48 ± 0.04) × 10^4^ s^−1^, respectively, which are all lower than that of wild-type Sdh2 enzyme. The mutant Q_D2_ more severely affects the catalytic activity in comparison to that of mutant Q_D1_. Their *K*_m_ values are 72.67 ± 5.73 and 53.54 ± 6.36 μM, respectively, approaching that of the wild-type Sdh2 enzyme. These data confirm that the identified Q_D1/2_ sites are the substrate-binding sites.

Complex II enzymes have been largely neglected for drug development in bacterial pathogens, despite them playing an essential role in bacterial metabolism and respiration^19^. Mammalian complex II is coupled to ubiquinone, whereas mycobacterial enzymes use menaquinone, providing opportunities for developing selective inhibitors. It has been reported that siccanin, a ubiquinone analog, can selectively inhibit the Sdhs from different species^38^, raising the possibility that menaquinone-based drugs can be specific against Sdhs and therefore be safe for use in humans. As for the Q_D1_ site (Fig. 3a), sequence alignments indicate the residues of quinone-binding site of Sdh2 protein from *M. tuberculosis* and *M. smegmatis* are highly similar, suggesting that they have a similar quinone-binding mechanism. However, despite the similarity of residues of the Q_D1_ sites between porcine and human, they are different from that of mycobacteria (Fig. 3c). Moreover, the Q_D1_-binding pocket in porcine SQR is formed by transmembrane helices IV, V and the N-terminal α-helix that stretches from the N terminus of transmembrane helix VI, but the binding pocket of Q_D1_ site *M. smegmatis* Sdh2 is involved in transmembrane helices IV, V and one bridging α-helix of them. This significant difference can be observed based on the superposition of their structures (Supplementary Fig. 4c). In terms of the Q_D2_ site (Fig. 3b), it is formed by the neighboring protomers, but the mammalian SQR exists only in monomeric form^6^ (Supplementary Fig. 4b). These observations thus suggest that the Q_D_ sites in *M. tuberculosis* are selective compared with those in the human counterpart. Thus, it is reasonable to suggest that this is a good antimycobacterial drug target, which is species selective.

### Electron transfer in the trimer offers clues to the cooperativity

**Fig. 4:**
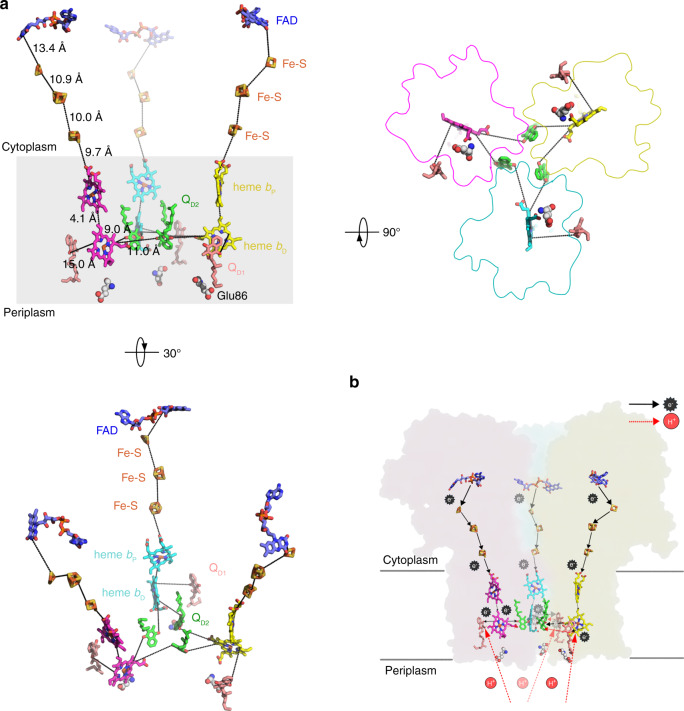
Electron/proton transfer pathway in the Sdh2 trimer. **a** The prosthetic groups, FAD, Fe-S clusters, hemes, and MK, are shown in stick or sphere. The edge-to-edge distances between adjacent prosthetic groups are labeled in black dashed lines. **b** Representation of electron and proton transfer way between adjacent protomers in the trimer. Electron transfer directions are shown in black arrows. Proton transfer pathways are shown by the red arrows.

In the *M. smegmatis* Sdh2 structure, the succinate oxidation site and MK reduction site are connected by a chain of redox centers including FAD, [2Fe-2S], [4Fe-4S], [3Fe-4S], *b*_P_ heme and *b*_D_ heme (Fig. 4a). *Bacillus* species SQRs also use diheme-containing SQRs^18^, and their *b*_D_ hemes are essential for electron transfer to menaquinone^35^. The EPR data (Supplementary Fig. 1e) also show that the electrons pass through *b*_D_ heme during menaquinone reduction. The distances between adjacent redox centers allow for efficient electron transfer^36^.

Previous studies have demonstrated that the monomeric and trimeric structure of the diheme-containing SQRs from *T. thermophiles* have SDH activities, but only the trimeric form exhibits positive cooperativity under high temperature^20,21^. The authors proposed that each monomer contains an MK binding site and can cooperatively interact with other MK-binding sites also exist within the trimeric structure. As described above, we have confirmed two substrate-binding sites (sites Q_D1_ and Q_D2_) by analyzing the catalytic activity of the mutant and wild-type Sdh2, and the features of Sdh2 structure. So, the structure here can provide a framework for understanding such a cooperative process. In addition, these data also demonstrate that the oxidized menaquinones bound to the Q_D1/2_ sites can accept the electrons from the *b*_D_ heme. But whether the menaquinone bound to the Q_D2_ site accesses to the electrons from the *b*_D_ hemes of adjacent monomers at the same time is unclear. It is worth noting that Sdh2 plays essential roles under hypoxia in *M. smegmatis*^23^ and *M. tuberculosis*^24^, suggesting that its trimeric nature may work cooperatively to meet the energy demand for survival or to effectively regulate energy metabolism under hypoxia^26^.

Therefore, the electrons are transferred from the succinate to menaquinone bound to Q_D1_ and Q_D2_ sites, achieving the oxidation of succinate and reduction of menaquinone (Fig. 4b). In *Bacillus subtilis*^17^ and *Bacillus lichenformis*^18^, it has been proposed that this type of reverse electron transport is driven by the proton potential, providing the driving force for MK reduction. As an analog of SQR, *W. succinogenes* QFR has corresponding pathways to proton transfer, in which Glu-C60 and Glu-C180 play crucial functional roles^14,15,39^. Although the present structure lacks an acidic residue at the equivalent position of Glu-C180, there is a well-conserved acidic residue Glu-D86 close to the Q_D1_ site, corresponding to Glu-C66 in *W. succinogenes* QFR^35^. This finding suggests that the Glu-D86 pathway participates in the proton transfer among the diheme-containing SQRs, and there is no canonical E-pathway-producing proton potential across the membrane. However, it has been proposed that one proton can be transferred directly from the quinol oxidation site to the Glu-C180 pathway in diheme-containing QFRs with the help of the ring C-propionate of *b*_D_ heme^40^, indicating the existence of a proton transfer pathway between the Q_D2_ site and ring C-propionate of *b*_D_ heme. Interestingly, the Q_D2_ site is located near the ring C-propionate of *b*_D_ heme of neighboring protomers in this structure, which suggests that during MK reduction at the Q_D2_ site protons may be uptaken through this pathway. Consequently, the Glu-D86 site is considered as the proton entry site, driving the reduction of MK molecules bound to Q_D1_ and Q_D2_ sites (Fig. 4b). Certainly, we cannot rule out the possibility of unidentified proton transfer pathways.

We have determined the cryo-EM structure of a trimeric Sdh from *M. smegmatis* at 2.8 Å, which identifies a non-covalently bound membrane-anchored subunit referred to as SdhF and shows that the electron transfer pathway is cooperative. Our study further provides a framework for rational structure-based anti-tuberculosis drug discovery.

## Methods

### Bacteria strain and culture

By means of homologous recombination, the Sdh2D gene (MSMEG_1671) was cloned into the expression plasmid pVV16 with a 10× His tag at the C terminus. *Mycobacterium smegmatis* mutant strain, mc^2^ 51^41^, was chosen for expression of the protein. The primers are listed in the Supplementary Table 4. Reconstructed cell solutions were cultured until the OD_600_ reached ~1.5 and then harvested by centrifugation for 30 min at 4000 r.p.m.

### Protein purification and characterization

Cells including the interest protein were lysed through a high-pressure cell disrupter at 4 °C and 1200 bar. The lysate was centrifuged at 14,000 r.p.m. for 10 min to remove cell debris and non-lysed cells. The resulting supernatant was centrifuged at 36,900 r.p.m. for 1 h in a Ti45 rotor (Beckman) and the membrane pellets were harvested. Membranes of the cells were extracted in buffer (20 mM MOPS, pH 7.4, 100 mM NaCl), and then stirred slowly at 4 °C for 3 h with the 1% (w/v) digitonin added. The supernatant after centrifugation was loaded onto a Ni-NTA column in the buffer containing 20 mM MOPS, pH 7.4, 100 mM NaCl, and 0.1% (w/v) digitonin, and the eluted fraction including the protein of interest was loaded onto a Superdex 200 (GE Healthcare) column equilibrated in a buffer containing 20 mM MOPS, pH 7.4, 100 mM NaCl and 0.1% (w/v) digitonin. The peak fractions were pooled and concentrated to 5 mg/mL.

To detect the protein components, the concentrated protein sample and the strips after SDS-PAGE analysis were subjected to MS analysis at the National Facility for Protein Science in Shanghai (NFPS). The protein sample was analyzed by BN-PAGE with NTB staining^42^. In order to use normal-phase liquid chromatography-MS (LCMS) to detect phospholipids and free mycolic acids^43,44^, we processed the samples as described previously study^27^. In addition, the levels of MK species were also quantified using a reverse-phase LCMS method^45^.

EPR spectra were acquired as previously described using a Bruker X-band (9.4 GHz) EMX plus 10/12 spectrometer with ESR-910 flowing helium cryostat, at a temperature of 15 K, and using a 5-Gauss modulation amplitude at 100 kHz under non-saturating MW power conditions (200 μW)^27^. To reveal different electron transfer components in the complex, samples in the “air-oxidized” state (as isolated) or “reduced” state (reduced by addition of 50 μM succinate or further reduced by 200 μM dithionite) were prepared^20,46–50^. The addition of reducing agents was done at room temperature followed by vortexing and freezing of the sample in liquid nitrogen within 5 min of addition.

### Characterization of enzyme activity

The protein samples were obtained by using plasmid pVV16 that is used for expression in *Mycobacterium smegmatis*, and the primers used in this study are listed in the Supplementary Table 4. The SQR activity of two different mutants Q_D1/2_ and wild-type of Sdh2 was determined using the DCIP assay according to a previous study^23^. In the present study, the menadione was selected as the intermediate electron acceptor. The reaction mixture contained 20 mM MOPS (pH 7.4), 0.005% LMNG (lauryl maltose neopentyl glycol), 0.025–1.6 mM sodium succinate, 1 mM menadione, and 100 μM DCIP. The reaction was initiated by the addition of DCIP into the mixture. All assays were performed at 37 °C, using a Molecular Devices iD3 Reader. Data were processed with GraphPad Prism 6.

### Cryo sample acquisition and data collection

Three microliters of aliquots of digitonin-solubilized protein at a concentration of 5 mg/mL were applied to H_2_/O_2_ glow-discharged 300-mesh Quantifoil R1.2/1.3 grids (Quantifoil, Micro Tools GmbH, Germany). Solution-absorbed grids were blotted for 2.5 s with force 1 at 8 °C and 100% humidity, and then plunged into pre-cooled liquid ethane using an FEI Vitrobot. Cryo-EM images were collected on a Titan Krios 300 kV electron microscope (Thermo Company), equipped with K3 Summit direct electron detector camera (Gatan). Images were recorded at ×29,000 magnification with a calibrated super-resolution pixel size 0.82 Å/pixel. The exposure time was set to 2 s with a total accumulated dose of 50 electrons per Å^2^. All images were automatically recorded using SerialEM^51^ with a defocus range from 1.2 to 2.2 μm. A total of 5420 images were collected.

### Image processing

All dose-fractioned images were motion-corrected and dose-weighted by the MotionCorr2 software^52^ and their contrast transfer functions were estimated by Gctf^53^. A total of 1,132,663 particles were picked automatically and extracted with a box size of 384 pixels in cryoSPARC^54^. The following 2D, 3D classifications and refinements were all performed in cryoSPARC^54^. A total of 564,244 particles were selected after two rounds of 2D classification. Hundred thousand particles were used to do Ab initio reconstruction in four classes, and then these four classes were used as 3D volume templates for heterogeneous refinement with all selected particles. In all, 461,385 particles converged into one class, yielding a 4.7 Å initial map. Then, this particle set was used to do homogeneous refinement, yielding 2.9 Å. After non-uniform refinement, the final resolution was 2.8 Å.

### Model building and refinement

An atomic model of Sdh2 was manually built in Coot 0.8.9.1^55^ using the crystal structure of porcine Sdh (PDB code: 1ZOY)^6^ as a template. From the local resolution map, the transmembrane domains had a relative high resolution, while the water-soluble subunits are at lower resolution in some regions. Thus, we were able to model correctly the side chains of the transmembrane subunits and the majority of water-soluble subunits. From the map and MS results, we were able to identify the relevant redox centers, phospholipids, MKs, and a subunit SdhF. All prosthetic groups and phospholipids models were generated using the elbow module in PHENIX with simple constraints^56^. These small molecules were docked into the map and refined in Coot 0.8.9.1. The differentiation of phospholipids is based on a previous study^27^. Final maps were sharpened automatically by applying a *B*-factor of 93.5 Å^2^ using cryoSPARC and subjected to real-space refinement using PHENIX. The local resolution map was calculated with ResMap^57^. All reported resolutions were based on the gold-standard FSC 0.143 criteria^58^. FSCwork and FSCtest were conducted to check for overfitting^59^.

All the figures were created by means of UCSF Chimera^60^ or PyMOL^61^.

### Reporting summary

Further information on research design is available in the Nature Research Reporting Summary linked to this article.

## Supplementary information

Supplementary Information

Reporting Summary

## Data Availability

The accession number for the 3D cryo-EM density map reported in present study is EMD-0981. The accession number for the coordinates for the structure reported in this study is 6LUM. Source data are provided with this paper.

## Source data

Source Data
