# Author Correction: Cryo-EM structure of trimeric *Mycobacterium smegmatis* succinate dehydrogenase with a membrane-anchor SdhF

**DOI:** 10.1038/s41467-021-21616-3

**Published:** 2021-02-23

**Authors:** Hongri Gong, Yan Gao, Xiaoting Zhou, Yu Xiao, Weiwei Wang, Yanting Tang, Shan Zhou, Yuying Zhang, Wenxin Ji, Lu Yu, Changlin Tian, Sin Man Lam, Guanghou Shui, Luke W. Guddat, Luet-Lok Wong, Quan Wang, Zihe Rao

**Affiliations:** 1 State Key Laboratory of Medicinal Chemical Biology, Frontiers Science Center for Cell Responses, College of Life Sciences, Nankai University, 300353 Tianjin, China; 2 Laboratory of Structural Biology, Tsinghua University, 100084 Beijing, China; 3 Shanghai Institute for Advanced Immunochemical Studies and School of Life Science and Technology, ShanghaiTech University, 201210 Shanghai, China; 4 National Laboratory of Biomacromolecules,omolecules, Institute of Biophysics, CAS, 100101 Beijing, China; 5 High Magnetic Field Laboratory, Chinese Academy of Sciences, 230031 Hefei, China; 6 Hefei National Laboratory of Physical Sciences at Microscale and School of Life Sciences, University of Science and Technology of China, 230027 Hefei, China; 7 State Key Laboratory of Molecular Developmental Biology, Institute of Genetics and Developmental Biology, CAS, 100101 Beijing, China; 8 School of Chemistry and Molecular Biosciences, The University of Queensland, Brisbane, 4072 QLD Australia; 9 Department of Chemistry, Inorganic Chemistry Laboratory, University of Oxford, Oxford, OX1 3QR UK

**Keywords:** Cryoelectron microscopy, Biophysics, Drug discovery, Microbiology

Correction to: *Nature Communications* 10.1038/s41467-020-18011-9, published online 25 August 2020.

The original version of this Article contained an error in the second last sentence of the Results and discussion section ‘Purification and characterization of *M. smegmatis* Sdh2’, where the Sdh2 apparent catalytic constant (*k*_*cat*_) value was incorrectly stated as (3.16 ± 0.07) × 10^4^ s^−1^, rather than the correct value of 3.16 ± 0.07 s^−1^. The original version of this Article also contained an error in the Results and discussion section ‘QD-binding site contributes to the structure-based drug discovery’, where the *k*_*cat*_ values of the QD1 and QD mutants were incorrectly stated as (2.16 ± 0.04) × 10^4^ s^−1^ and (1.48 ± 0.04) × 10^4^ s^−1^, which has been corrected to 2.16 ± 0.04 s^−1^ and 1.48 ± 0.04 s^−1^, respectively. This has been corrected in both the PDF and HTML versions of the Article.

