# Supplementary material for: Cryo-EM structure of trimeric Mycobacterium smegmatis succinate dehydrogenase with a membrane-anchor SdhF
Source: Nat Commun. 2020 Aug 25;11:4245. doi: 10.1038/s41467-020-18011-9 (PMC7447783; doi:10.1038/s41467-020-18011-9)
Supplement: Supplementary file 1 — Supplementary Information [file 41467_2020_18011_MOESM1_ESM.pdf]

Supplementary Information

Cryo-EM structure of trimeric *Mycobacterium smegmatis* succinate dehydrogenase with a membrane-anchor SdhF

Hongri Gong, Yan Gao et al.

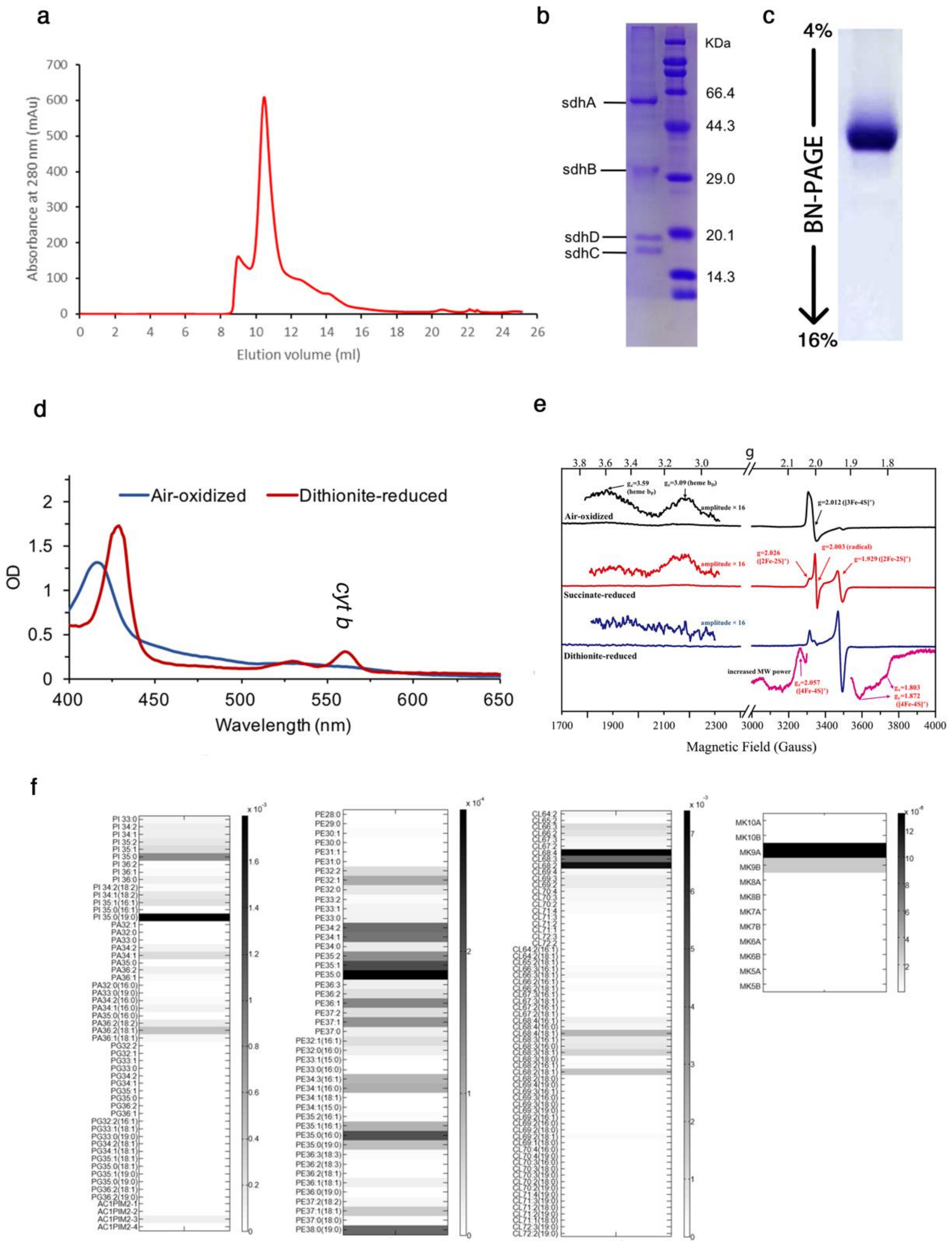

Supplementary Fig. 1 Purification and component identification of the Sdh2 trimer from *Mycobacterium smegmatis*. a, The elution profile of the Sdh2 trimer from size exclusion chromatography (SEC). Peak fractions were pooled and concentrated for preparation on the cryo-EM grids. **b,** SDS-PAGE of the pooled fraction from SEC in **a.** Each band is labelled with the corresponding subunit, which were then identified by mass spectrometry

(Table S1). **c,** Blue Native PAGE of the pooled fraction from SEC in **a**. The gel was stained with PMS/NTB. **d,** UV-visible spectra of the dithionite-reduced (black) and air-oxidized (red) forms of the Sdh2 trimer. The reduced wavelengths correspond to absorbance peaks of the cytochrome *b* (563 nm). **e,** EPR characterization of the purified complex. EPR spectra of the air-oxidized (black trace), succinate-reduced (red trace) and dithionite-reduced (blue and purple trace) complex. The EPR spectrum of the air-oxidized sample (black trace) showing characteristic peaks corresponding to the g_z of the two highly anisotropic low spin (HALS)-type heme groups and the oxidized $[3Fe\text{-}4S]^+$ center; The EPR spectrum of the succinate-reduced sample (red trace) showing peaks arising from the reduced $[2Fe\text{-}2S]^+$ cluster with an additional peak centered at g=2.003 attributable to a semiquinone (SQ) radical. Part of the amplitude of signal corresponding to heme b_P was lost upon reduction by succinate; Further reduction with dithionite (blue trace) resulted in complete loss of signal for both hemes due to complete reduction, along with the appearance of peaks from reduced $[4Fe\text{-}4S]^+$ when the increased microwave (MW) power was increased to 15 mW (purple trace). Spectra were scaled correspondingly to highlight different metal centers. The central signals of dithionite-reduced samples under high MW power have been removed to highlight the outer g-values associated with the signal of $[4Fe\text{-}4S]^+$. **f,** The identification of lipids and variants of MKs in the protein sample.

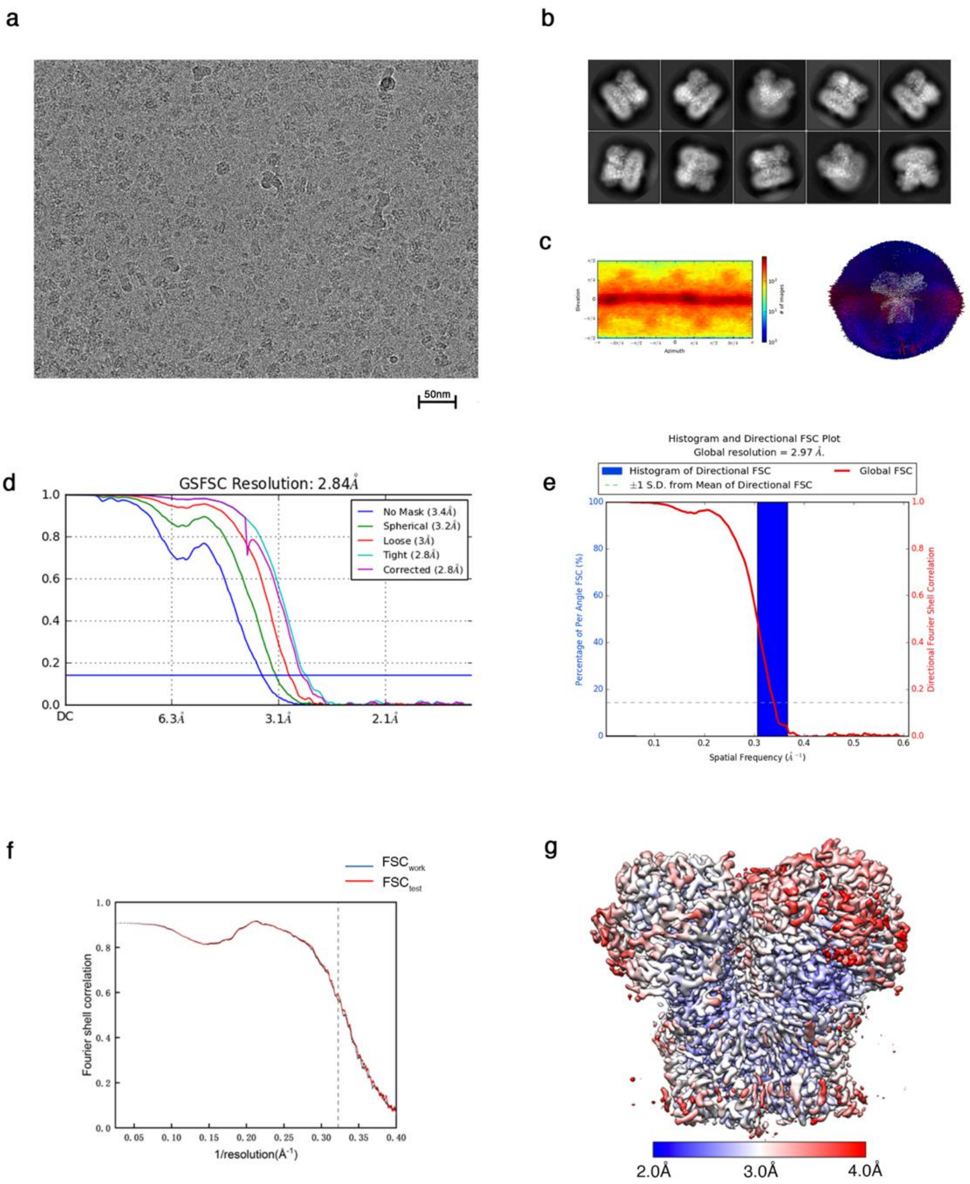

Supplementary Fig. 2 Cryo-EM structure of the *M. smegmatis* Sdh2 complex. a, Representative electron micrograph of the cryo-EM sample. **b,** Representative 2D classification averages calculated from selected particles. **c,** Viewing direction and Eeuler angle distributions of all particles used in the final 3D reconstruction. **d,** FSC curves of 3D reconstructions. **e,** 3DFSC histogram of final map. **f,** FSC_{work} and FSC_{test} calculations. **g,** Front view of the density map, which is colored according to the local resolution.

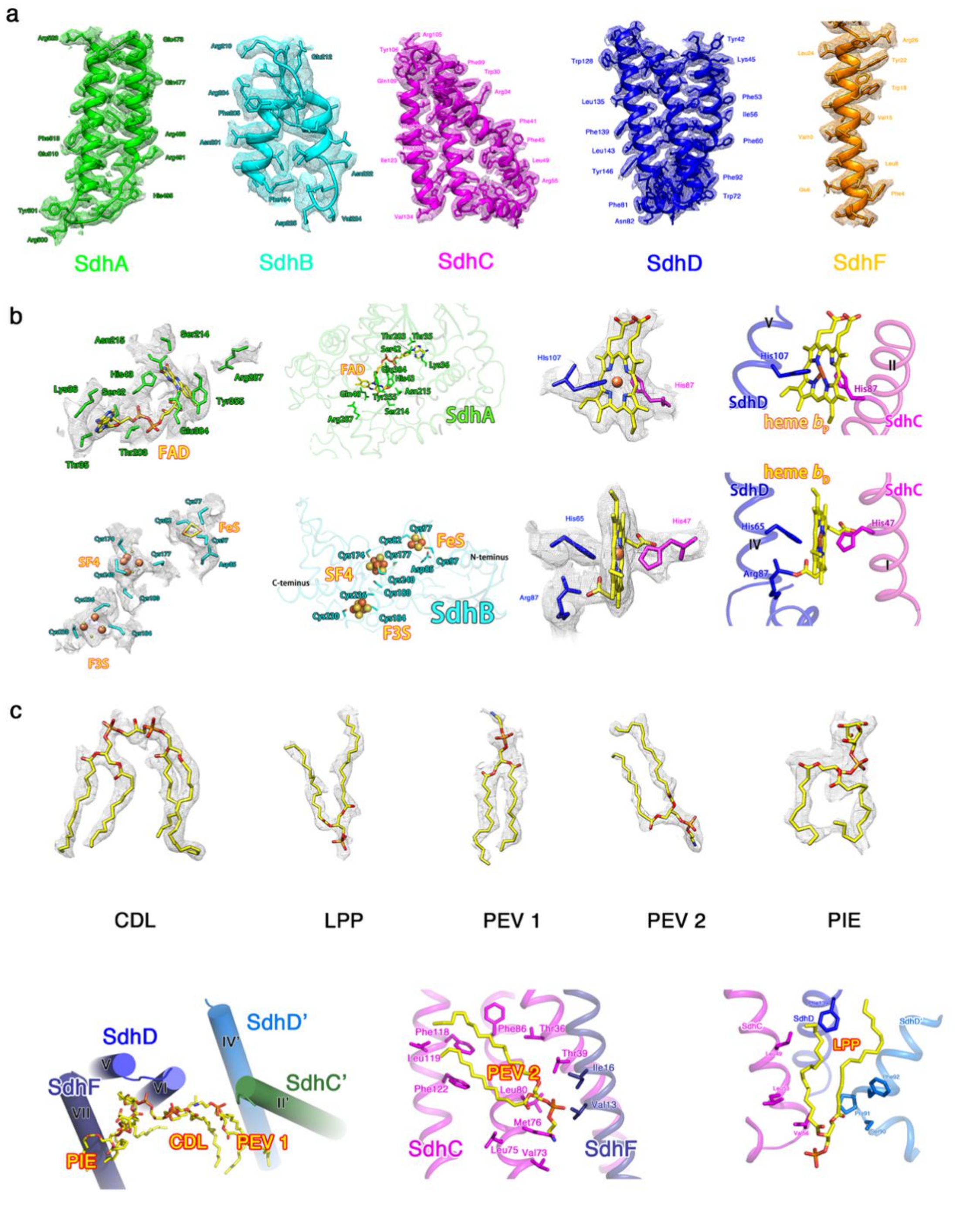

Supplementary Fig. 3 Cryo-EM map quality assessment and ligand representation of Sdh2 protein. Representative cryo-EM densities of **(a)** individual subunits, **(b)** prosthetic groups and **(c)** phospholipids of the Sdh2 trimer from *M. smegmatis*. Corresponding subunits or residues interacting with prosthetic groups and phospholipids are shown in stick or cartoon representation.

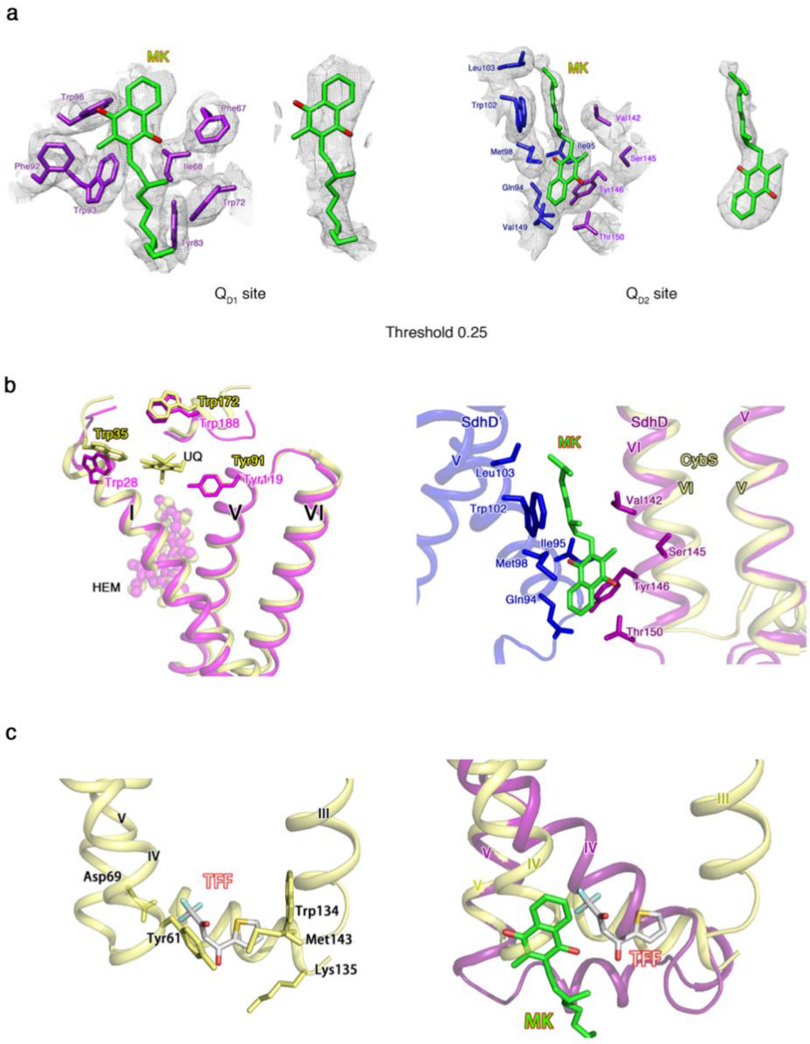

Supplementary Fig. 4 Quinone sites in *M. smegmatis* Sdh2 and porcine SQR. a, Representative cryo-EM densities of menaquinone/menaquinol molecules bound in *M. smegmatis* Sdh2. Individual densities for each MK is shown. **b,** Structural superposition between *M. smegmatis* Sdh2 (purple) and porcine SQR (yellow) with bound UQ1 (PDB: 1ZOY). Canonical Qp (left) and newly identified Q_{D2} sites (right) are shown. For the Q_{D2} site, the neighboring SdhD subunit of another monomer is colored in blue. **c,** (left) Q_{D1} binding pocket of porcine SQR with bound TFF inhibitor (PDB: 1ZP0). (right) Structural superposition of Q_{D1} site for *M. smegmatis* Sdh2 and porcine SQR.

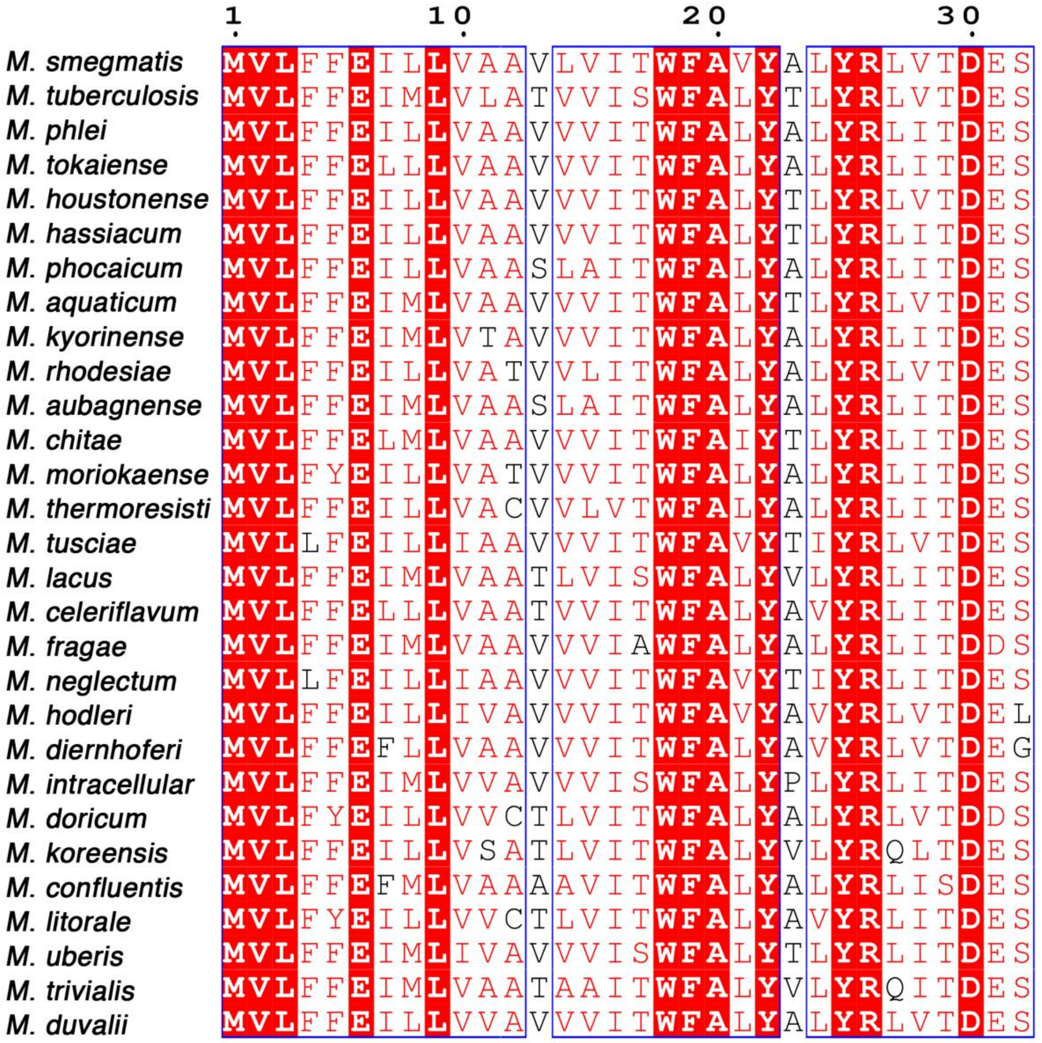

Supplementary Fig. 5 Sequence alignments of SdhF subunits. In order to evaluate the conservation of the subunit SdhF in the *phylum Actinobacteria*, homologous sequences were retrieved through the NCBI BLAST. The highly homologous sequences of representative species of mycobacteria were obtained.

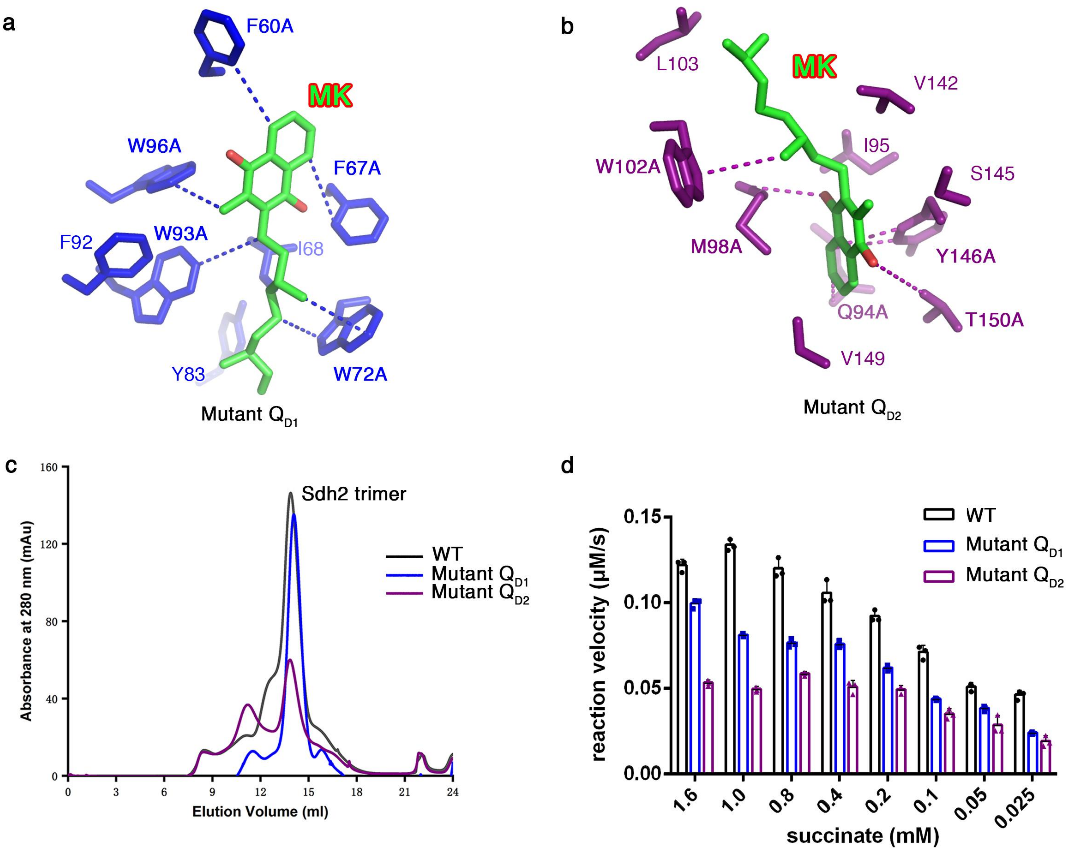

Supplementary Fig. 6 Characterization of activities of the wild-type and mutant Sdh2 complex. Residues potentially interacting with menaquinone in (**a**) Q_{D1} site and (**b**) Q_{D2} site are labeled. The residues, Phe60, Phe67, Trp72, Trp93, and Trp96 from Q_{D1} site and Qln94, Met98, Trp102, Tyr146, and Thr150 from Q_{D2} site were mutated to alanine, respectively. (**c**) Gel filtration chromatography for wt and two mutants. (**d**) Enzymatic activity for the wild-type, mutated Q_{D1} and mutated Q_{D2} of Sdh2 complexes. Mean values resulting from technical triplicates and error bars using standard deviation are shown.

Supplementary Table 1. Identification of the Sdh2 complex subunits by peptide mass fingerprint spectroscopy

Subunits			Mass Spectroscopy	
Protein accession	Protein Name	Theoretical Molecular Weight (kDa)	Peptides	Coverage
WP_011727811.1	SdhA	64.4	45	81
WP_003893076.1	SdhB	29.2	26	76
WP_011727812.1	SdhC	15.6	16	83
WP_003893078.1	SdhD	17.9	15	90
WP_011730792.1	SdhF	3.7	1	100

Supplementary Table 2. Statistics for data collection, image processing and model building

	M. smegmatis Sdh2 complex
**PDB entry**	6LUM
**EMDB entry**	EMD-0981
**Data collection and processing**	
Magnification	29,000
Voltage (keV)	300
Electron exposure ($e^{-}/Å^2$)	50.00
Defocus range (μm)	-1.2 to -2.2
Pixel size (Å)	0.82
Symmetry imposed	C1
Initial particle images (no.)	1,973,698
Final particle images (no.)	461,385
Map global resolution (Å)	2.84
Global resolution FSC threshold	0.143
Map local resolution range (Å)	2.0-8.0
Local resolution FSC threshold	0.5
**Refinement**	
Model resolution (Å)	2.8
FSC threshold	0.143
Model resolution range (Å)	to 2.8
Map sharpening *B* factor ($Å^2$)	93.5
Model composition	
Non-hydrogen atoms	26,191
Protein residues	3,178
Ligands	39
B factors ($Å^2$)	
Protein	30.42
Ligand	35.82
R.m.s. deviations	
Bond lengths (Å)	0.010
Bond angles (°)	1.165
Validation	
MolProbity score	2.09
Clashscore	10.26
Poor rotamers (%)	0.08
Ramachandran plot	
Outliers	0.13
Allowed	10.18
Favored	89.69
EMRinger score	3.31
Model vs. Data	
CC(mask)	0.86
CC(volume)	0.81
Mean CC for ligands	0.80

Supplementary Table 3. Summary of the model

Subunit Name	Chain	Total residues/range built	% atomic model	Cofactors	Resolution (Å)
**SdhA**	A/J/P	2-305, 347-581/<br>2-300, 347-581/<br>2-305, 347-381	92.0	FAD	2.5~6.0
**SdhB**	B/K/Q	23-260/14-261/23-260	92.5	2Fe-2S,<br>3Fe-3S,<br>4Fe-4S	2.0~4.0
**SdhC**	C/G/M	15-136/15-137/16-138	88.9	Heme b	2.0~3.5
**SdhD**	D/H/N	11-156/37-156/41-156	81.6	Heme b	2.0~3.5
**SdhF**	E/I/O	1-31	96.9		2.5~3.5

Supplementary Table 4. Primers used in molecular cloning

SdhD-F	5'-gccaagacaattgcggatccgtgagcgcgccaggggcagg-3'
SdhD-R	5'-tggtgatgatggtgatggtgtgaaatattcgcgtcgaagg-3'
pVV16-F	5'-ggatccgcaattgtcttggc-3'
pVV16-R	5'-caccatcaccatcatcacca-3'
Mutant_Q_{D1}_60-F	5'-tggccctggcgctcggccacc-3'
Mutant_Q_{D1}_60-R	5'-cagggccaccagcgcaatgcc-3'
Mutant_Q_{D1}_67_72-F	5'-gccatcatgctgatggcgcaggacggcgtgtaccggat-3'
Mutant_Q_{D1}_67_72-R	5'-cgccatcagcatgatggccaggtggccgagcgccagg-3'
Mutant_Q_{D1}_93_96-F	5'-ttcgcgcagatcgcggacatggcc-3'
Mutant_Q_{D1}_93_96-R	5'-tccgcgatctgcgcgaacggcgagg-3'
Mutant_Q_{D2}_94-F	5'-gcgatctgggacatggccctgctctgg-3'
Mutant_Q_{D2}_94-R	5'-gtcccagatcgcccagaacggcgaggc-3'
Mutant_Q_{D2}_98-F	5'-gcggccctgctctggttggcaatgatc-3'
Mutant_Q_{D2}_98-R	5'-gagcagggccgcgtcccagatcgccca-3'
Mutant_Q_{D2}_102-F	5'-gcgttggcaatgatccacggcgccaac-3'
Mutant_Q_{D2}_102-R	5'-cattgccaacgcgagcagggccgcgtc-3'
Mutant_Q_{D2}_146_150-F	5'-gcagcgccgtactggtcgccttcgacgcgaata-3'
Mutant_Q_{D2}_146_150-R	5'-tcgaaggcgaccagtacggcgctgcccagcaccaac-3'
